# Endovascular thrombectomy without versus with different pre-intravenous thrombolysis in acute ischemic stroke: a network meta-analysis of randomized controlled trials

**DOI:** 10.3389/fneur.2024.1344961

**Published:** 2024-01-29

**Authors:** Sitong Guo, Shiran Qin, Sitao Tan, Henghai Su, Xiaoyu Chen

**Affiliations:** Department of Pharmacy, Guangxi Academy of Medical Sciences and the People’s Hospital of Guangxi Zhuang Autonomous Region, Nanning, China

**Keywords:** acute ischemic stroke, endovascular thrombectomy, tenecteplase, alteplase, network meta-analysis

## Abstract

**Background:**

The current guideline recommended the use of intravenous thrombolysis (IVT) before Endovascular thrombectomy (EVT), but the effectiveness and safety of tenecteplase compare to alteplase in patients before EVT remain uncertain.

**Methods:**

We searched PubMed, Embase, Web of Science, and the Cochrane Library to identify eligible articles from inception until September 16, 2023. The primary outcome was functional independence (mRS 0–2) at 90 days. Secondary outcomes included excellent outcome (mRS 0–1) at 90 days, all-cause mortality at follow-up, successful reperfusion (TICI 2b–3) after the end of EVT, symptomatic intracranial hemorrhage (sICH) or any intracranial hemorrhage (aICH). The PROSPERO registration number is CRD42023470419.

**Results:**

Eight randomized controlled trials (RCTs) were included involving 2,836 acute ischemic stroke (AIS) patients. Compared to EVT alone, tenecteplase (0.25 mg/kg and 0.4 mg/kg) + EVT and 0.9 mg/kg alteplase + EVT were significant difference associated with higher successful reperfusion (TICI 2b–3) after the end of EVT (RR = 2.31; 95% CI 1.15–4.63; RR = 2.31; 95% CI 1.00–5.33; RR = 1.05; 95% CI 1.01–1.09). And compared to 0.25 mg/kg tenecteplase + EVT, alteplase (0.6 mg/kg and 0.9 mg/kg) + EVT were significant difference associated with lower successful reperfusion (TICI 2b–3) after the end of EVT (RR = 0.45; 95% CI 0.22–0.90; RR = 0.45; 95% CI 0.23–0.91). The risk of aICH (RR = 1.50; 95% CI 1.07–2.09) was significantly higher for 0.6 mg/kg alteplase + EVT than EVT alone. There was no significant difference in functional independence (mRS 0–2), excellent outcome (mRS 0–1), all-cause mortality or sICH among the different IVT strategies (0.25 mg/kg or 0.4 mg/kg tenecteplase and 0.6 mg/kg or 0.9 mg/kg alteplase) before EVT.

**Conclusion:**

The use of alteplase before EVT may potentially improve the successful reperfusion after EVT compared to tenecteplase. Due to the insufficient sample size, more high-quality RCTs are needed to confirm effectiveness and safety of tenecteplase compare to alteplase in patients before EVT.

**Systematic review registration:**

https://www.crd.york.ac.uk/prospero/, identifier: CRD42023470419.

## Background

Large vessel occlusions are often a significant contributing factor to the worsening of neurological function in patients with acute ischemic stroke (AIS) (1). Endovascular thrombectomy (EVT) can rapidly restore blood flow and improve clinical outcomes, which has attracted significant attention from everyone (2). Prior to the implementation of EVT in clinical settings, intravenous thrombolysis (IVT) using alteplase (ALT) and tenecteplase (TNK) was the sole treatment available for AIS patients with large vessel occlusions (AIS-LVO) within the designated treatment time frame (3). For AIS-LVO patients with no contraindications to thrombolysis, current guidelines recommend the use of intravenous thrombolysis followed by EVT (Class I recommendation) (4). However, there is still some controversy surrounding the benefits and risks of IVT prior to EVT, and it has not been definitively established in clinical practice. Although recent meta-analyses have suggested potential benefits of IVT as a pretreatment for EVT (5–7), several observational studies have yielded conflicting results in terms of favorable functional outcomes (8–13) or mortality rates at 90 days (10, 11). The potential benefits of IVT before EVT may be attributed to facilitating thrombus dislodgement, enhancing collateral circulation, or aiding in the dissolution of distal thrombi that cannot be accessed by the endovascular device (14–16). However, these hypotheses have not been supported by three recently published randomized controlled trials (RCTs) (17–19) and a prospective cohort study (20).

In addition, tenecteplase has gradually emerged as a promising alternative for the clinical treatment of AIS through IVT. Compared to alteplase, it has a longer half-life, greater specificity for fibrin, and lower affinity for plasminogen activator inhibitor (PAI) (21, 22). Currently, the majority of clinical studies have used alteplase for IVT before EVT. However, there is a lack of direct comparison studies on the effectiveness and safety of alteplase and tenecteplase before EVT. Therefore, we included RCTs and conducted a systematic review and network meta-analysis (NMA) to compare the effectiveness and safety of tenecteplase with alteplase for thrombolysis before EVT.

## Materials and methods

This study has been registered on the PROSPERO platform (https://www.crd.york.ac.uk/prospero/) Registration number: CRD42023470419, and the study followed the PRISMA statement.

### Data sources and search

The databases of PubMed, Embase, Web of Science, and the Cochrane Library were retrieved to identify studies that evaluated the effects of nafamostat in ECMO from inception until September 16, 2023, without language restrictions. The search strategy included three key concepts according to the PICOS format: (“endovascular thrombectomy” OR “mechanical thrombectomy” OR “intravenous thrombolysis” OR “tenecteplase” OR “alteplase”) AND “acute ischemic stroke” AND “human” AND “randomized controlled trial” (Supplementary Table S1). Medical Subject Headings and relevant keywords were used to identify potential articles. The search strategy is shown in Supplementary Table S2. The references of pertinent studies were screened to identify additional studies.

Two authors independently searched the databases, excluded duplicates, read titles and abstracts, and thoroughly evaluated the full texts to select potentially qualified research. A third author participated in the discussion and resolution of disagreement.

### Study selection

The inclusion criteria were the following: (1) patients with AIS-LVO; (2) randomized controlled trials (RCTs); (3) comparing EVT alone versus thrombectomy with pre-IVT or comparing thrombectomy with different pre-IVT in ischemic stroke; and (4) evaluation of functional independence (mRS 0–2) at 90 days, excellent outcome (mRS 0–1) at 90 days, all-cause mortality at follow-up, successful reperfusion (TICI 2b–3) after the end of EVT, symptomatic intracranial hemorrhage (sICH) or any ICH outcomes. Exclusion criteria were: (1) studies lacking a control group; (2) non-cohort studies like case reports, cross-sectional studies, etc.; (3) studies assessing irrelevant outcomes; and (4) studies with incomplete primary data records unsuitable for statistical analysis and where comprehensive information could not be obtained from authors.

### Data extraction and outcomes

A pre-specified form was used to extract information from the included studies. The following information was collected: publication year, study design, subject characteristics (age and sex), sample size, intervention and control protocol and outcomes. Two authors independently extracted the data, and all extracted information was cross-checked for accuracy by a third author.

Primary outcome was functional independence (mRS 0–2) at 90 days. Secondary outcomes included excellent outcome (mRS 0–1) at 90 days, all-cause mortality at follow-up, successful reperfusion (TICI 2b–3) after the end of EVT, sICH and any ICH.

### Quality assessment

We strictly adopted the standardized criteria for assessing the risk of bias in RCTs as established by the Cochrane Collaboration. We utilized Review Manager (RevMan) 5.3 software to assess the risk of bias in each RCT, considering seven aspects: selection bias, performance bias, detection bias, attrition bias, reporting bias, and other potential biases. The classification for each bias criterion was categorized as “low,” “high,” or “unclear.”

### Statistical analysis

This study conducted analyses from two aspects: comparing the effectiveness and safety of different pre-IVT strategies before EVT (network meta-analysis), and comparing the effectiveness and safety of EVT with and without pre-IVT (meta-analysis). STATA 15.0 (Stata Corporation, College Station) and Review Manager 5.3 were used to perform meta-analysis and network meta-analysis. Relative risk (RR) and 95% confidence intervals (CI) of the outcomes were calculated and pooled in the random- or fixed-effect model, depending on the heterogeneity between studies. An *I^2^ test* of >50% indicated significant heterogeneity. The fixed-effects model was used based on the generic inverse variance method unless significant heterogeneity was present. A value of *p* < 0.05 was considered statistically significant for all analyses.

For network meta-analysis (EVT alone versus 0.25 mg/kg TNK/0.4 mg/kg TNK/0.6 mg/kg ALT/0.9 mg/kg ALT + EVT), to perform multivariate random-effects network meta-analysis and indirect comparisons within a frequency analysis framework, we utilized the STATA command “mvmeta.” Due to the absence of a closed loop in the network diagram, no inconsistency test was conducted. The network diagram was created using the “network graph” feature in STATA. Each node in the graph represents a specific vascular reperfusion intervention, and the size of the nodes as well as the thickness of the connecting lines represent the number of studies involving the intervention and the number of head-to-head studies between two interventions. We calculated the Surface Under the Cumulative Ranking (SUCRA) curve to assess the relative rankings of the intervention measures. This ranking displays the cumulative probability of each intervention measure being the best or worst treatment option. The magnitude of SUCRA is positively correlated with the effectiveness of the intervention measures.

For meta-analysis (EVT alone versus IVT + EVT), IVT include 0.25 mg/kg TNK, 0.6 mg/kg ALT or 0.9 mg/kg ALT, trial sequential analysis (TSA) was conducted to assess the reliability of discontinuous variable and determine if the sample size was adequate. When the Z-score curve (blue line) crossed the boundary of statistical significance (red dashed line) and intersected above the solid green line, it indicated that the results were reliable and the sample size was sufficient. Sensitivity analysis of the primary and secondary outcomes was performed using the leave-one-out method to assess the reliability of the results. Meta-regression analysis was applied to detect factors that affect outcome indicators by including the demographic characteristics of the study.

## Results

### Study selection

**Figure 1:**
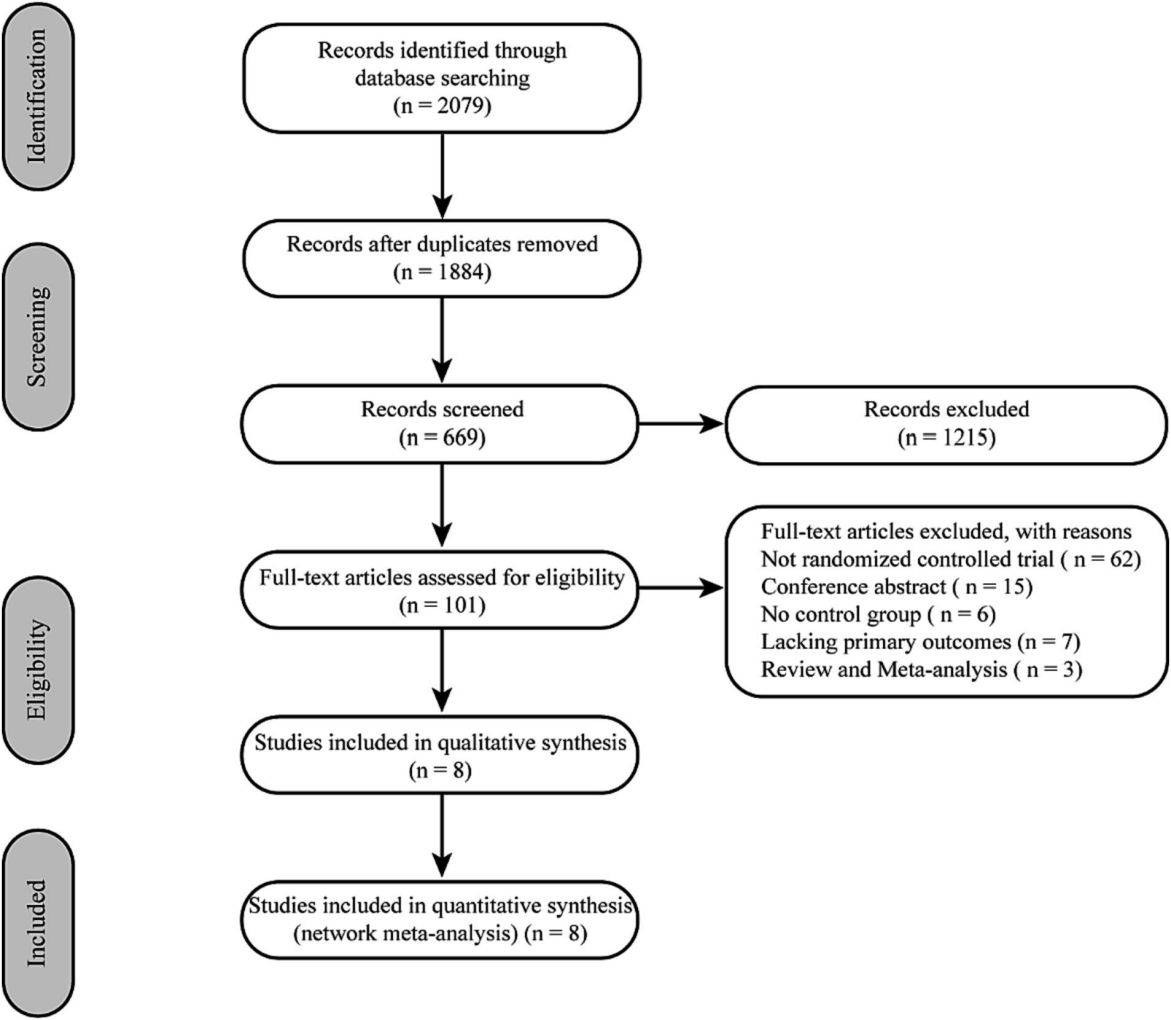
Flow diagram of study selection.

Initially, 2079 records from electronic databases were screened through a comprehensive search. After removing 195 duplicates and screening the titles and abstracts of the remaining records, 1,215 studies were excluded. Eight RCTs were included after the full article assessment (17–19, 23–27) (Figure 1).

### Characteristics of the included trials

**Figure 2:**
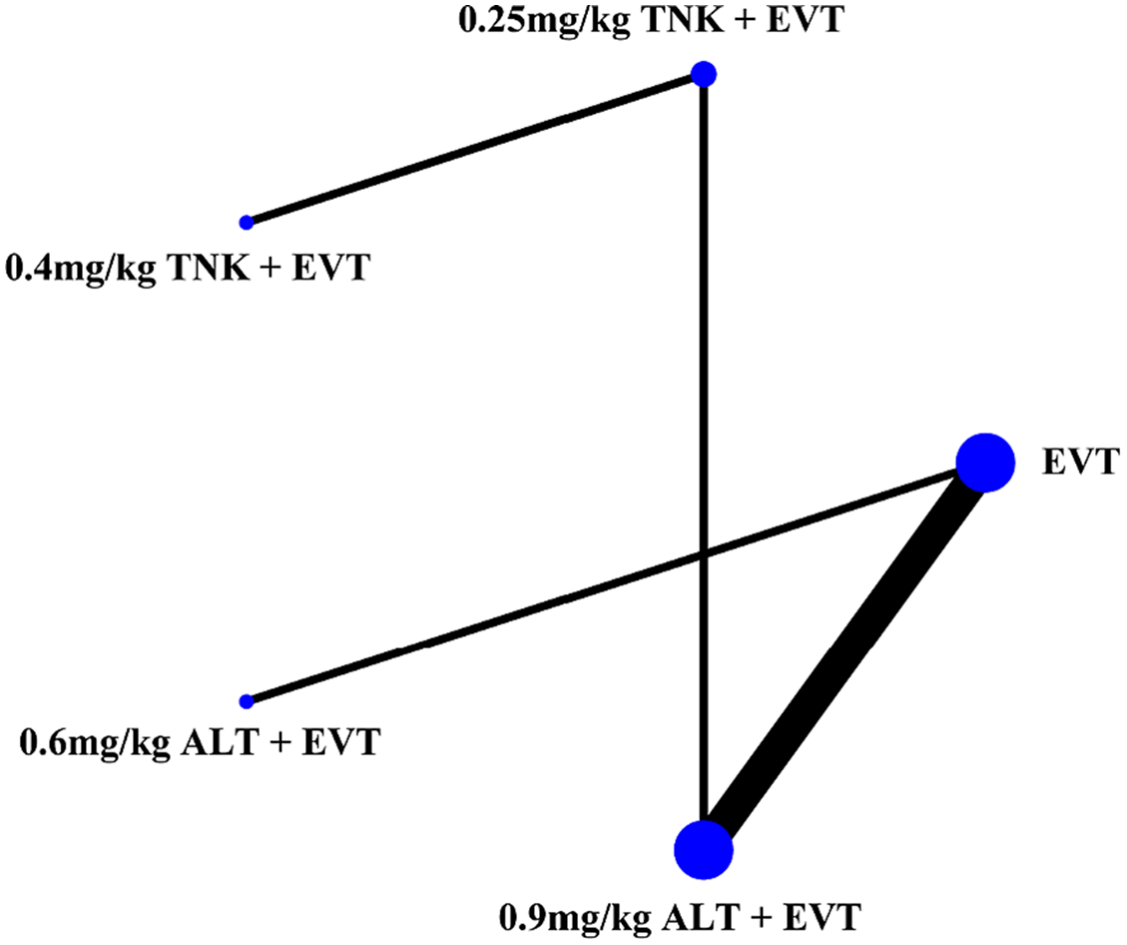
Network graph of compared treatment strategies. ALT, alteplase; EVT, endovascular thrombectomy; TNK, Tenecteplase.

Eight RCTs with 2,836 AIS patients were included. The patients were divided into 5 treatment groups according to the treatment strategies they received: EVT alone, 0.25 mg/kg TNK + EVT, 0.4 mg/kg TNK + EVT, 0.6 mg/kg ALT + EVT and 0.9 mg/kg ALT + EVT. Of the 2,836 participants, 1,599 were men and 1,237 were women, ranging from 69.0 to 86.6 years old. A total of 8 studies reported functional independence (mRS 0–2) at 90 days, all-cause mortality at follow-up, successful reperfusion (eTICI 2b–3) and sICH. Excellent outcome (mRS 0–1) was reported in 7 records that included 2,428 cases, and 6 records reported any ICH (Table 1). Network graph of compared treatment strategies, show as (Figure 2).

**Table 1:** Characteristics of the included trials.

Study (first author, year)	Country	Study design	Time period	T/C	Sample size (M/F)	Age	Intervention and control protocol		Occlusion vessel	Outcomes
							Intervention	Control		
(23) (EXTEND-IA TNK Part 1)	Australia and New Zealand	RCT	2015–2018	101/101	202 (110/92)	T: 70.4 ± 15.1 C: 71.9 ± 13.7	Tenecteplase, 0.25 mg/kg + MT	Alteplase, 0.9 mg/kg + MT	AC/PC	a, b, c, d, e
(24) (EXTEND-IA TNK Part 2)	Australia and New Zealand	RCT	2017–2019	150/150	300 (159/141)	T: 71.7 ± 11.3 C: 73.8 ± 12.8	Tenecteplase, 0.4 mg/kg + MT	Tenecteplase, 0.25 mg/kg + MT	AC/PC	a, b, c, d, e
(17) (SKIP)	Japan	RCT	2017–2019	103/101	204 (158/76)	T: 76.0 C: 74.0	Alteplase, 0.6 mg/kg + MT	MT	AC	a, b, c, d, e, f
(18) (DIRECT-MT)	China	RCT	2018–2019	329/327	656 (370/286)	T: 69.0 C: 69.0	Alteplase, 0.9 mg/kg + MT	MT	AC	a, b, c, d, e, f
(19) (DEVT)	China	RCT	2018–2020	118/116	234 (132/102)	T: 70.0 C: 70.0	Alteplase, 0.9 mg/kg + MT	MT	AC	a, b, c, d, e, f
(26) (MR CLEAN–NO IV)	Netherlands, Belgium, and France	RCT	2020–2021	266/273	539 (305/234)	T: 69.0 C: 72.0	Alteplase, 0.9 mg/kg + MT	MT	AC	a, b, c, d, e, f
(25) (SWIFT DIRECT)	Europe and Canada	RCT	2017–2021	207/201	408 (199/209)	T: 72.0 C: 73.0	Alteplase, 0.9 mg/kg + MT	MT	AC	a, c, d, e, f
(27) (DIRECT-SAFE)	Australia, New Zealand, China, and Vietnam	RCT	2018–2021	147/146	293 (166/127)	T: 69.0 C: 70.0	Alteplase (83%), 0.9 mg/kg OR Tenecteplase (17%), 0.25 mg/kg + MT	MT	AC/PC	a, b, c, d, e, f

a, functional independence (mRS 0–2) at 90 days; b, excellent outcome (mRS 0–1) at 90 days; c, all-cause mortality at follow-up; d, successful reperfusion (TICI 2b–3) after the end of EVT; e, symptomatic intracranial hemorrhage; f, any intracranial hemorrhage. AC, indicates anteror circuaton; EVT, endovascular thrombectomy; M/F, Male/female; mRS, modified Rankin Scale; PC, posterior circulation; T/C, treatment group/control group; TICI, Thrombolysis in Cerebral Ischemia; RCT, randomized controlled trial.

### Risk of bias

As shown in Supplementary Figure 1, the bias risk and methodological quality of the included 8 RCTs were assessed using the Cochrane bias risk assessment tool. Apart from six studies that indicated a high risk of performance bias, the risk of other biases was low, indicating that the overall quality of the included studies is high.

### Functional independence (mRS 0–2)

**Figure 3:**
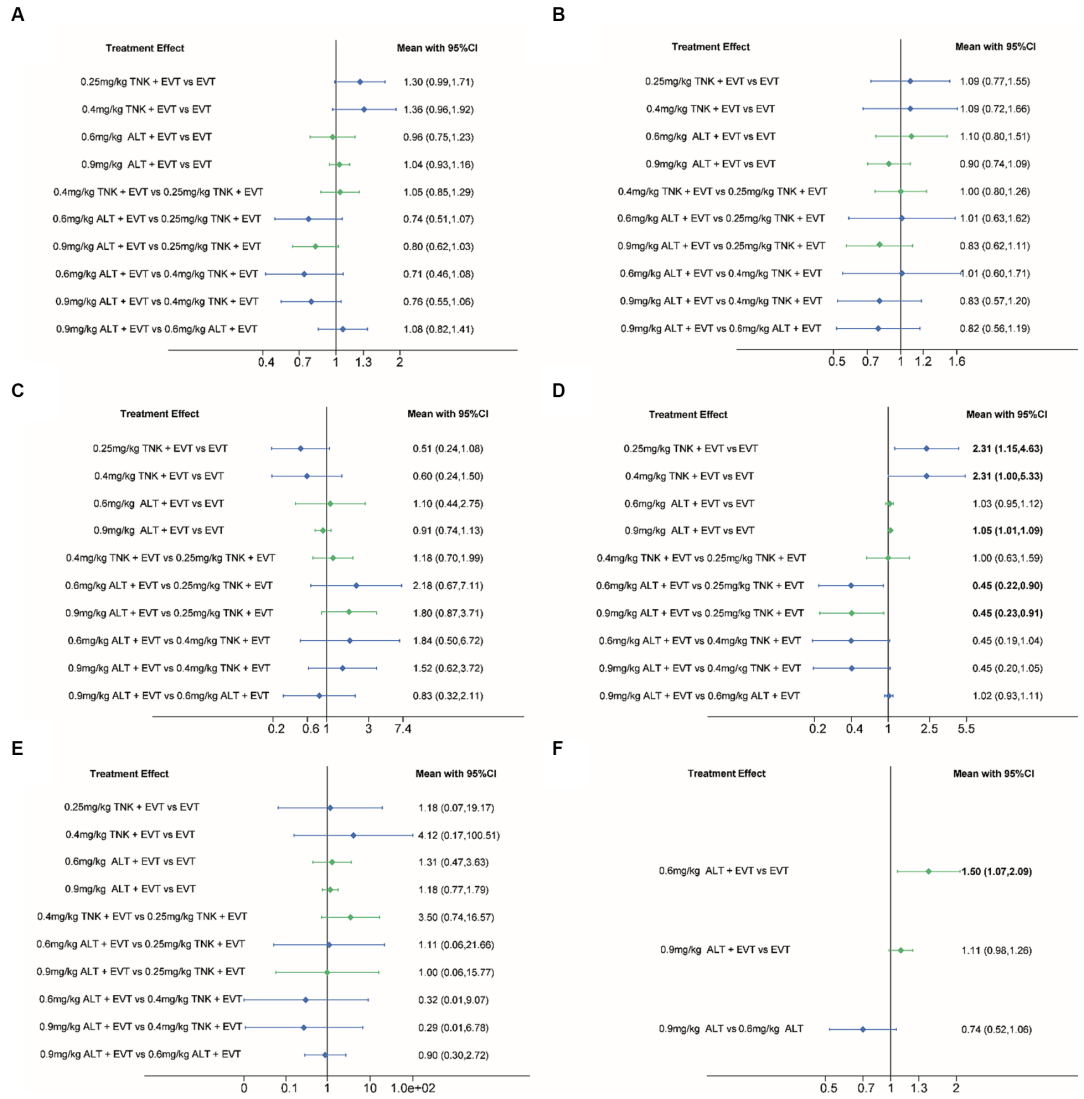
Forest plot for the network meta-analysis of all outcomes. Pooled RRs and 95% CIs determined by network meta-analysis, where direct comparisons (green) and indirect comparisons (blue) are shown. **(A)** Functional independence (mRS 0–2) at 90 days; **(B)** excellent outcome (mRS 0–1) at 90 days; **(C)** all-cause mortality at follow-up; **(D)** successful reperfusion (TICI 2b–3) after the end of EVT; **(E)** symptomatic intracranial hemorrhage; **(F)** any intracranial hemorrhage. CI, confidence interval; ALT, alteplase; EVT, endovascular thrombectomy; mRS, modified Rankin Scale; RR, Relative risk; TNK, Tenecteplase; TICI, Thrombolysis in Cerebral Ischemia.

**Figure 4:**
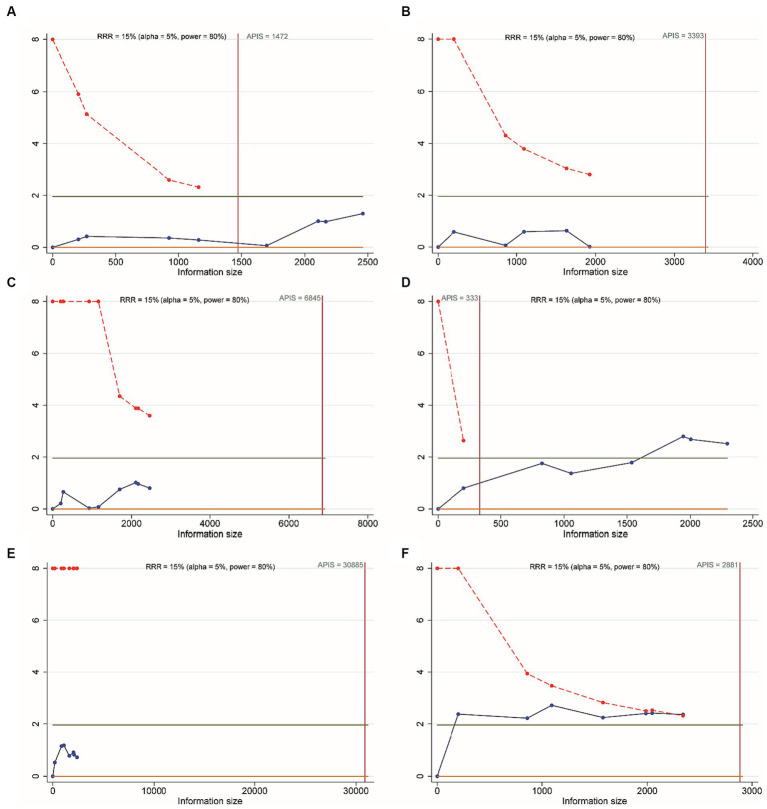
Trial sequential analysis. **(A)** Functional independence (mRS 0–2) at 90 days; **(B)** excellent outcome (mRS 0–1) at 90 days; **(C)** all-cause mortality at follow-up; **(D)** successful reperfusion (TICI 2b–3) after the end of EVT; **(E)** symptomatic intracranial hemorrhage; **(F)** any intracranial hemorrhage.

All included RCTs accounted for functional independence (mRS 0–2) and there were seven RCTs with 2,543 participants included in the NMA. Compared to EVT alone, 0.25 mg/kg TNK + EVT, 0.4 mg/kg TNK + EVT, 0.6 mg/kg ALT + EVT or 0.9 mg/kg ALT + EVT were not associated with significant difference in functional independence (mRS 0–2) at 90 days (Figure 3A). Additionally, the SUCRA values showed that 0.6 mg/kg ALT + EVT ranked first (SUCRA 80.6%), followed by EVT (SUCRA 76.8%), 0.9 mg/kg ALT + EVT (SUCRA 60.6%), 0.25 mg/kg TNK + EVT (SUCRA 20.0%) and 0.4 mg/kg TNK + EVT (SUCRA 12.0%) (Table 2). Compared to EVT alone, IVT + EVT (IVT include 0.25 mg/kg TNK or 0.6 mg/kg ALT or 0.9 mg/kg ALT) was not significantly associated with a better functional independence (mRS 0–2) at 90 days (Supplementary Figure S2A; RR = 1.04; 95% CI 0.96–1.12, *p* = 0.46). Trial sequential analysis showed that the accumulated amount of information has exceeded the expected amount. The lack of statistical difference between the two groups suggests that the efficacy of the EVT alone and IVT + EVT can be considered similar in functional independence (mRS 0–2) at 90 days (Figure 4A).

**Table 2:** The surface under the cumulative ranking (SUCRA).

Intervention	mRS (0–2)			mRS (0–1)			All-cause mortality			TICI			sICH			aICH		
	SUCRA (%)	PrBest	MR	SUCRA (%)	PrBest	MR	SUCRA (%)	PrBest	MR	SUCRA (%)	PrBest	MR	SUCRA (%)	PrBest	MR	SUCRA (%)	PrBest	MR
EVT	76.8	28.5	1.9	54.1	9.0	2.8	24.1	0.8	4.0	93.8	76.4	1.2	70.6	31.0	2.2	97.3	94.6	1.1
0.25 mg/kg TNK + EVT	20.0	1.0	4.2	36.0	4.0	3.6	88.4	63.6	1.5	13.3	0.6	4.5	60.7	39.4	2.6			
0.4 mg/kg TNK + EVT	12.0	1.7	4.5	39.1	13.5	3.4	69.1	24.8	2.2	14.8	2.3	4.4	18.5	3.4	4.3			
0.6 mg/kg ALT + EVT	80.6	58.4	1.8	34.6	11.5	3.6	26.4	0.3	3.9	7.0	20.4	2.2	48.7	18.1	3.1	3.1	0.9	2.9
0.9 mg/kg ALT + EVT	60.6	10.4	2.6	86.2	62.0	1.6	42.2	2.6	3.3	58.2	0.3	2.7	51.4	8.1	2.9	49.6	4.5	2.0

ALT, alteplase; aICH, any intracranial hemorrhage; EVT, endovascular thrombectomy; mRS, modified Rankin Scale; SUCRA, the surface under the cumulative ranking; sICH, symptomatic intracranial hemorrhage; TNK, Tenecteplase; TICI, Thrombolysis in Cerebral Ischemia.

### Excellent outcome (mRS 0–1)

Six RCTs accounted for excellent outcome (mRS 0–1) with 2,135 participants included in the NMA. Compared to EVT alone, 0.25 mg/kg TNK + EVT, 0.4 mg/kg TNK + EVT, 0.6 mg/kg ALT + EVT or 0.9 mg/kg ALT + EVT were not associated with significant difference in excellent outcome (mRS 0–1) at 90 days (Figure 3B). Additionally, the SUCRA values showed that 0.9 mg/kg ALT + EVT ranked first (SUCRA 86.2%), followed by 0.4 mg/kg TNK + EVT (SUCRA 54.1%), EVT (SUCRA 39.1%), 0.25 mg/kg TNK + EVT (SUCRA 36.0%) and 0.6 mg/kg ALT + EVT (SUCRA 34.6%) (Table 2). Compared to EVT alone, IVT + EVT was not significantly associated with a better excellent outcome (mRS 0–1) at 90 days (Supplementary Figure S2B; RR = 0.99; 95% CI 0.86–1.13, *p* = 0.58). Trial sequential analysis showed the accumulated amount of information not reaching the expected level, it is possible that there is no difference between EVT alone and IVT + EVT in excellent outcome (mRS 0–1) at 90 days. However, further experiments are needed to provide more evidence and confirm this (Figure 4B).

### All-cause mortality

For the NMA, seven RCTs reported all-cause mortality during follow-up. In comparison with EVT alone, 0.25 mg/kg TNK + EVT, 0.4 mg/kg TNK + EVT, 0.6 mg/kg ALT + EVT or 0.9 mg/kg ALT + EVT were not associated with significant difference in all-cause mortality (Figure 3C). Additionally, the SUCRA values showed that 0.25 mg/kg TNK + EVT ranked first (SUCRA 88.4%), followed by 0.4 mg/kg TNK + EVT (SUCRA 69.1%), 0.9 mg/kg ALT + EVT (SUCRA 42.2%), 0.6 mg/kg ALT + EVT (SUCRA 26.4%) and EVT (SUCRA 24.1%) (Table 2). Compared to EVT alone, IVT + EVT was not significantly associated with a lower all-cause mortality (Supplementary Figure S2C; RR = 0.99; 95% CI 0.78–1.13, *p* = 0.74). Trial sequential analysis showed the accumulated amount of information not reaching the expected level, it is possible that there is no difference between EVT alone and IVT + EVT in all-cause mortality during follow-up. However, further experiments are needed to provide more evidence and confirm this (Figure 4C).

### Successful reperfusion (TICI 2b–3)

For the NMA, seven RCTs reported successful reperfusion (TICI 2b–3) after the end of EVT. In comparison with EVT alone, 0.25 mg/kg TNK + EVT, 0.4 mg/kg TNK + EVT and 0.9 mg/kg ALT + EVT were significant difference associated with higher successful reperfusion (TICI 2b–3) after the end of EVT (Figure 3D; RR = 2.31; 95% CI 1.15–4.63; RR = 2.31; 95% CI 1.00–5.33; RR = 1.05; 95% CI 1.01–1.09). Additionally, compared to 0.25 mg/kg TNK + EVT, 0.6 mg/kg ALT + EVT and 0.9 mg/kg ALT + EVT were significant difference associated with lower successful reperfusion (TICI 2b–3) after the end of EVT (Figure 3D; RR = 0.45; 95% CI 0.22–0.90; RR = 0.45; 95% CI 0.23–0.91). There is no significant difference observed in pairwise comparisons between other intervention measures. The SUCRA values showed that EVT ranked first (SUCRA 93.8%), followed by 0.9 mg/kg ALT + EVT (SUCRA 58.2%), 0.4 mg/kg TNK + EVT (SUCRA 14.8%), 0.25 mg/kg TNK + EVT (SUCRA 13.3%) and 0.6 mg/kg ALT + EVT (SUCRA 7.0%) (Table 2). Compared to EVT alone, IVT + EVT was significantly associated with a higher successful reperfusion (TICI 2b–3) (Supplementary Figure S2D; RR = 1.04; 95% CI 1.01–1.08, *p* = 0.69). It is worth noting that, when compared to EVT alone, the TSA results for TICI 2b-3 in IVT + EVT suggest a lack of conclusive evidence regarding this endpoint. This is because the cumulative z-curve only crosses the conventional boundaries rather than the monitoring boundary of the trial sequence (Figure 4D).

### Symptomatic intracranial hemorrhage (sICH)

Seven RCTs accounted for symptomatic intracranial hemorrhage (sICH) included in the NMA. In comparison with EVT alone, 0.25 mg/kg TNK + EVT, 0.4 mg/kg TNK + EVT, 0.6 mg/kg ALT + EVT or 0.9 mg/kg ALT + EVT were not associated with significant difference in sICH (Figure 3E). Additionally, the SUCRA values showed that EVT ranked first (SUCRA 70.6%), followed by 0.25 mg/kg TNK + EVT (SUCRA 60.7%), 0.6 mg/kg ALT + EVT (SUCRA 51.4%), 0.6 mg/kg ALT + EVT (SUCRA 48.7%) and 0.4 mg/kg TNK + EVT (SUCRA 18.5%) (Table 2). Compared to EVT alone, IVT + EVT was not significantly associated with a lower sICH (Supplementary Figure S2E; RR = 0.99; 95% CI 0.86–1.13, *p* = 0.58). Trial sequential analysis showed the accumulated amount of information not reaching the expected level, it is possible that there is no difference between EVT alone and IVT + EVT in sICH. However, further experiments are needed to provide more evidence and confirm this (Figure 4E).

### Any intracranial hemorrhage (aICH)

Five RCTs accounted for any intracranial hemorrhage (ICH) included in the NMA. In comparison with EVT alone, 0.6 mg/kg ALT + EVT was significant difference associated with any sICH (Figure 3F; RR = 1.50; 95% CI 1.07–2.09). There is no significant difference observed in pairwise comparisons between other intervention measures. Additionally, the SUCRA values showed that EVT ranked first (SUCRA 97.3%), followed by 0.9 mg/kg ALT + EVT (SUCRA 49.6%) and 0.6 mg/kg ALT + EVT (SUCRA 3.1%) (Table 2). Compared to EVT alone, IVT + EVT was significantly associated with a lower sICH (Supplementary Figure S2F; RR = 1.15; 95% CI 1.02–1.28, *p* = 0.34). Trial sequential analysis showed the cumulative amount of information has not reached the expected value, but the *Z*-value of the statistical test has crossed the threshold. Therefore, further experiments are not necessary, and we can draw a conclusive conclusion in advance (Figure 4F).

### Sensitivity analysis and meta-regression analysis

The leave-one-out sensitivity analysis did not indicate significant variation in the primary and secondary outcomes (Supplementary Figure S3). Meta-regression analysis was conducted to confirm the impact of possible confounding factors included in the analysis on functional independence at 90 days, excellent outcome at 90 days, all-cause mortality at follow-up, successful reperfusion after the end of EVT, sICH and any ICH (Supplementary Table S3).

### Publication bias

**Figure 5:**
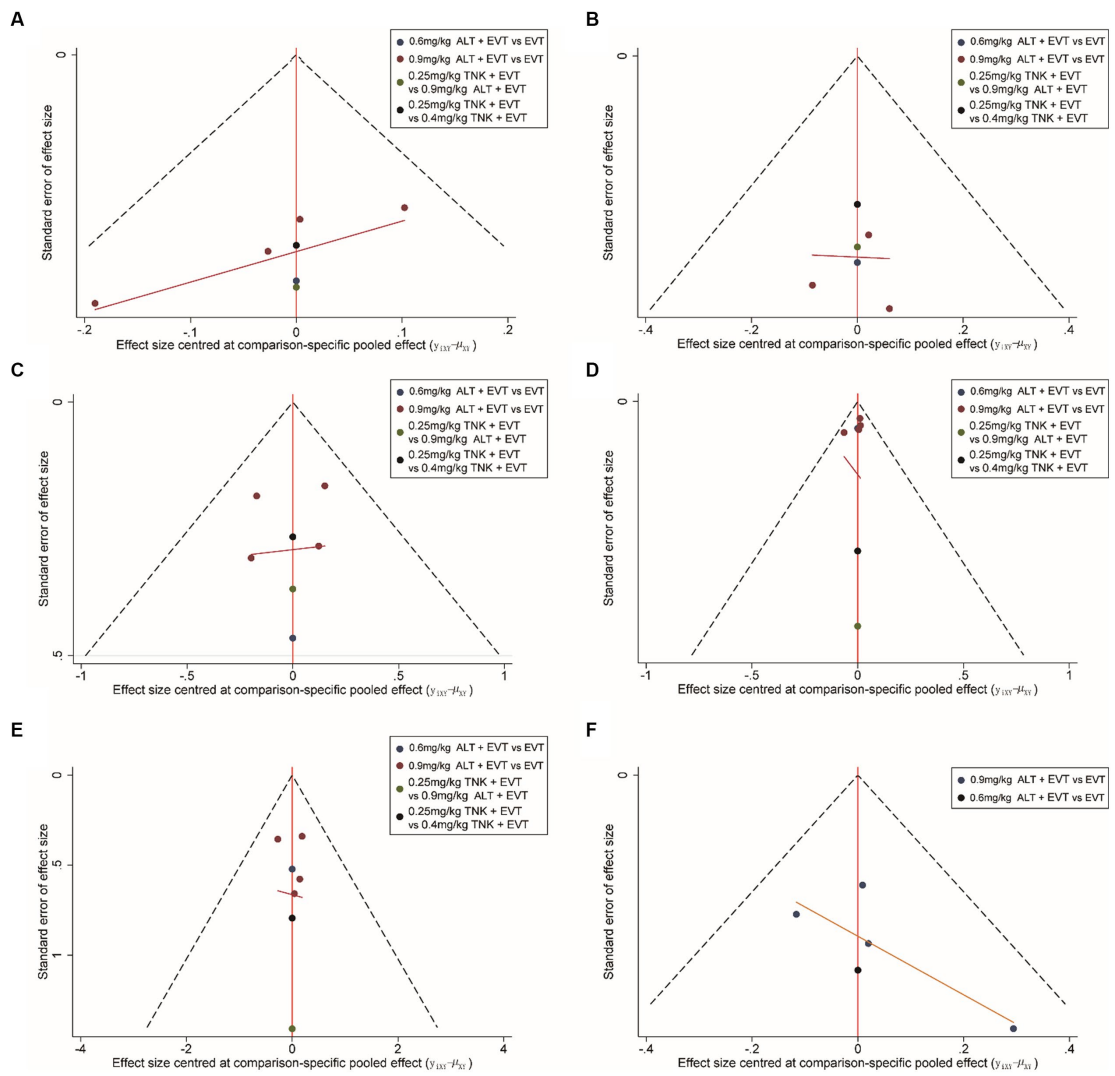
The publication bias analysis. **(A)** Functional independence (mRS 0–2) at 90 days; **(B)** excellent outcome (mRS 0–1) at 90 days; **(C)** all-cause mortality at follow-up; **(D)** successful reperfusion (TICI 2b–3) after the end of EVT; **(E)** symptomatic intracranial hemorrhage; **(F)** any intracranial hemorrhage. ALT, alteplase; EVT, endovascular thrombectomy; TNK, Tenecteplase.

We utilized the method of comparison-adjusted funnel plot to assess publication bias and small-study effects. The evaluation of whether the funnel plot is approximately symmetrical suggests no evidence of publication bias (Figure 5). However, it is important to acknowledge that the assessment of publication bias using funnel plots could be somewhat unreliable for clinical outcomes with limited inclusion of studies.

## Discussion

The main findings of this NMA study regarding whether to perform IVT before EVT and the different pre-intravenous thrombolysis strategies include data from 8 RCTs involving 2,836 patients are as follows: (1) successful reperfusion (TICI 2b–3): compared to EVT alone, the rate of successful reperfusion was higher with 0.25 mg/kg TNK + EVT, 0.4 mg/kg TNK + EVT and 0.9 mg/kg ALT + EVT. Additionally, compared to 0.25 mg/kg TNK + EVT, 0.6 mg/kg ALT + EVT, and 0.9 mg/kg ALT + EVT, the rate of successful reperfusion was higher. Furthermore, compared to EVT alone, IVT + EVT had a higher rate of successful reperfusion. The comparison results between 0.9 mg/kg ALT + EVT and 0.25 mg/kg TNK + EVT were based on direct comparison, while the remaining comparisons were based on indirect comparison results; (2) any intracranial hemorrhage: the direct comparison results suggest that there is a higher risk of intracranial hemorrhage associated with the use of 0.6 mg/kg ALT + EVT compared to EVT alone; and (3) no significant difference was observed for functional independence (mRS 0–2), excellent outcome (mRS 0–1), all-cause mortality and sICH with EVT alone, 0.25 mg/kg TNK + EVT, 0.4 mg/kg TNK + EVT, 0.6 mg/kg ALT + EVT, 0.9 mg/kg ALT + EVT and IVT + EVT.

The main clinical symptoms of acute ischemic stroke are speech weakness, limb weakness, mouth skew, confusion, coma and so on (28, 29). If it is not treated in time, the blocked blood vessels can not be recanalized in time, which can lead to irreversible damage to the neurological function of the body. At present, the specific pathogenesis of acute ischemic stroke is not very clear, in which platelet adhesion, massive release of inflammatory factors and thrombosis play an important role in the occurrence and development of the disease (30–32). Nowadays, it is generally believed that dredging and embolizing blood vessels as soon as possible, improving the reversible injury of brain cells to promote the recovery of cerebral blood supply, and saving the ischemic penumbra around the infarcted area to the maximum extent is the key to the treatment of acute ischemic stroke (33). In recent years, mechanical thrombectomy has been gradually used in clinic, which can quickly promote the recanalization of obstructed blood vessels and obtain better curative effect in acute ischemic stroke (34, 35).

Although some current evidence suggests the use of IVT before EVT, the immediate transfer of AIS patients to a medical center with endovascular capabilities or receiving IVT at the nearest facility after symptom onset is crucial and may have a direct impact on patient prognosis. Therefore, the utility and necessity of using IVT before EVT remain a topic of intense debate (7). Moreover, existing studies primarily assess the effects of intravenous thrombolysis with alteplase before EVT compared to EVT alone on establishing reperfusion. Due to the lack of comparison between the use of tenecteplase and alteplase before EVT, the effectiveness and safety of tenecteplase prior to EVT have not garnered much attention.

To our knowledge, this study is the first attempt to analyze the effectiveness and safety of four IVT strategies, include 0.25 mg/kg TNK, 0.4 mg/kg TNK, 0.6 mg/kg ALT and 0.9 mg/kg ALT, before EVT. The results of successful reperfusion (TICI 2b–3) after EVT indicate that the use of 0.6 mg/kg ALT or 0.9 mg/kg ALT before EVT is superior to 0.25 mg/kg TNK, while 0.25 mg/kg TNK, 0.4 mg/kg TNK and 0.9 mg/kg ALT are superior to EVT alone. Based on the above results, it appears that the use of ALT before EVT is superior to 0.25 mg/kg TNK. However, due to the small sample size and lack of direct comparison, the results should be interpreted with caution. Similarly, in the comparison between EVT alone and IVT + EVT, the successful reperfusion rate in IVT + EVT is significantly higher than EVT alone. However, the sequential analysis results suggest a lack of conclusive evidence regarding this endpoint, so caution is still needed in interpreting the results. The mechanism by which pre-IVT improves the rate of successful reperfusion is not yet clear, as IVT may not completely dissolve large clots. However, studies have shown that common intraoperative thrombus fragments occur after EVT, leading to downstream microvascular embolization or thrombus formation, obstructing distal arterial flow. Therefore, the potential increase in vascular recanalization rate with the use of IVT before EVT may be related to its ability to separate the clot surface from the vascular endothelium, making it easier to retrieve and aspirate (36, 37).

This NMA showed that 0.6 mg/kg ALT + EVT and IVT (0.6 mg/kg ALT or 0.9 mg/kg ALT or 0.25 mg/kg TNK) + EVT has a significantly higher incidence of aICH compared to EVT alone, but functional independence (mRS 0–2), excellent outcome (mRS 0–1), all-cause mortality and sICH were not different. Trial sequential analysis showed the cumulative amount of information has not reached the expected value, but further experiments are not necessary, and we can draw a conclusive conclusion in advance. It is worth noting that among the 6 RCTs included in this study for aICH analysis, only a small portion of patients in one RCT used 0.25 mg/kg TNK as the intravenous thrombolytic agent. The potential significant difference in the incidence of aICH between tenecteplase and alteplase prior to EVT remains unknown, further clinical studies are needed to verify this.

The present NMA indicated that no significant difference was observed for functional independence (mRS 0–2), excellent outcome (mRS 0–1), all-cause mortality and sICH with EVT alone, 0.25 mg/kg TNK + EVT, 0.4 mg/kg TNK + EVT, 0.6 mg/kg ALT + EVT, 0.9 mg/kg ALT + EVT and IVT + EVT. However, the results of the TSA analysis comparing EVT with IVT + EVT suggest that, except for the outcome measure of functional independence, the cumulative amount of information for the other outcome measures did not meet the expected threshold. In other words, with more clinical studies conducted in the future, the results of meta-analysis may differ.

This study has the following limitations: (1) all included RCTs had slightly different assessment methods for defining sICH; (2) the absence of closed loops in the network diagram indicates that the consistency of all study and analysis results supports the robustness of the conclusion; (3) the NMA has fewer than 10 studies, and although a publication bias analysis was conducted, the effectiveness of the test may be relatively low; and (4) data (less than 10 studies) were insufficient for meta-regression in the outcome, and the results of heterogeneity source analysis may not be reliable. Therefore, the results should be considered cautiously since the possibility of confounders must be considered.

## Conclusion

In conclusion, the available RCT evidence suggests that the outcomes of ALT + EVT and TNK + EVT are similar, compared to tenecteplase, the pre-EVT use of alteplase may potentially improve the successful reperfusion after EVT. And compared with EVT alone, current evidence showed that IVT + EVT demonstrated higher successful reperfusion and aICH, with caution needed in interpreting the advantage of successful reperfusion while the risk of aICH remains robust. Due to the insufficient sample size, although other outcome measures did not show significant differences, with more high-quality clinical studies emerging, significant differences may potentially arise.

## Data availability statement

The original contributions presented in the study are included in the article/Supplementary material, further inquiries can be directed to the corresponding authors.

## Author contributions

SG: Conceptualization, Writing – original draft. SQ: Data curation, Writing – original draft. ST: Software, Writing – original draft. HS: Data curation, Formal analysis, Writing – original draft. XC: Writing – review & editing.
