# Supplementary material for: Endovascular thrombectomy without versus with different pre-intravenous thrombolysis in acute ischemic stroke: a network meta-analysis of randomized controlled trials
Source: Front Neurol. 2024 Jan 29;15:1344961. doi: 10.3389/fneur.2024.1344961 (PMC10860706; doi:10.3389/fneur.2024.1344961)
Supplement: Supplementary file 1 [file Data_Sheet_1.pdf]

Endovascular thrombectomy without versus with different pre-intravenous thrombolysis in acute ischemic stroke: a network meta-analysis of randomized controlled trials

Supplementary materials

Supplementary Figure 1. Risk of bias of included studies. (A) Risk of bias summary: judgments about each bias item for each study; (B) Risk of bias summary graph.

Supplementary Figure 2. Forest plot of subgroups analysis of outcomes. (A) functional independence (mRS 0–2) at 90 days; (B) excellent outcome (mRS 0–1) at 90 days; (C) all-cause mortality at follow-up; (D) successful reperfusion (TICI 2b–3) after the end of EVT; (E) symptomatic intracranial hemorrhage; (F) any intracranial hemorrhage.

Supplementary Figure 3. Sensitivity analysis with leave-one-out method for outcomes. (A) functional independence (mRS 0–2) at 90 days; (B) excellent outcome (mRS 0–1) at 90 days; (C) all-cause mortality at follow-up; (D) successful reperfusion (TICI 2b–3) after the end of EVT; (E) symptomatic intracranial hemorrhage; (F) any intracranial hemorrhage.

Supplementary Figure 4. Contribution plot of outcomes. (A) functional independence (mRS 0–2) at 90 days; (B) excellent outcome (mRS 0–1) at 90 days; (C) all-cause mortality at follow-up; (D) successful reperfusion (TICI 2b–3) after the end of EVT; (E) symptomatic intracranial hemorrhage; (F) any intracranial hemorrhage.

Supplementary Table 1. PICOS format for the research question.

Supplementary Table 2. Search strategy for the included databases based.

Supplementary Table 3. Meta-regression analysis of outcomes.

Supplementary Table 4. The results of Egger and Begg.

Supplementary Table 1. PICOS format for the research question

**P**: Acute ischemic stroke patients
**I**: intravenous thrombolysis before Endovascular thrombectomy
**C**: Endovascular thrombectomy alone
**O**: Functional independence (mRS 0–2) at 90 days or Excellent outcome (mRS 0–1) at 90 days or All-cause mortality at follow-up or Successful reperfusion (TICI 2b–3) after the end of EVT or Symptomatic intracranial hemorrhage (sICH) or Any intracranial hemorrhage (aICH)
**S**: RCTs

Supplementary Table 2. Search strategy for the included databases based (PubMed as an example)

PubMed (September 16, 2023)		
**Number**	**Searches**	**Results**
#1	"recombinant tissue plasminogen activator"[Title/Abstract] OR "alteplase"[Title/Abstract] OR "rt-PA"[Title/Abstract]	7,473
#2	(("tenecteplase"[MeSH Terms] OR "TNK-tPA"[Title/Abstract] OR "tenecteplase"[Title/Abstract]) OR (Metalyse[Title/Abstract])) OR (TNKase[Title/Abstract])	876
#3	(((((((((((((((((((((((((((Ischemic Strokes[MeSH Terms])) OR Ischemic Stroke*[Title/Abstract]) OR (Stroke, Ischemic[Title/Abstract])) OR (Ischaemic Stroke[Title/Abstract])) OR (Ischaemic Strokes[Title/Abstract])) OR (Stroke, Ischaemic[Title/Abstract])) OR (Cryptogenic Ischemic Stroke[Title/Abstract])) OR (Cryptogenic Ischemic Strokes[Title/Abstract])) OR (Ischemic Stroke, Cryptogenic[Title/Abstract])) OR (Stroke, Cryptogenic Ischemic[Title/Abstract])) OR (Cryptogenic Stroke[Title/Abstract])) OR (Cryptogenic Strokes[Title/Abstract])) OR (Stroke, Cryptogenic[Title/Abstract])) OR (Cryptogenic Embolism Stroke[Title/Abstract])) OR (Cryptogenic Embolism Strokes[Title/Abstract])) OR (Embolism Stroke, Cryptogenic[Title/Abstract])) OR (Stroke, Cryptogenic Embolism[Title/Abstract])) OR (Wake-up Stroke[Title/Abstract])) OR (Stroke, Wake-up[Title/Abstract])) OR (Wake up Stroke[Title/Abstract])) OR (Wake-up Strokes[Title/Abstract])) OR (Acute Ischemic Stroke[Title/Abstract])) OR (Acute Ischemic Strokes[Title/Abstract])) OR (Ischemic Stroke, Acute[Title/Abstract])) OR (Stroke, Acute Ischemic[Title/Abstract])) OR (cerebral arterial thrombosis[Title/Abstract])) OR (ischemic cerebral	78,425

	apoplexy[Title/Abstract])	
#4	(((((((("Thrombectomy"[Mesh]) OR ("Embolectomy"[Mesh])) OR (Thrombectomy[Title/Abstract])) OR (Thrombectomies[Title/Abstract])) OR (Percutaneous Aspiration Thrombectomy[Title/Abstract])) OR (Aspiration Thrombectomies, Percutaneous[Title/Abstract])) OR (endovascular thrombectomy[Title/Abstract])) OR (mechanical thrombectomy[Title/Abstract])) OR (Embolectomy[Title/Abstract])) OR (Embolectomies[Title/Abstract])	23,827
#5	(((((((randomized controlled trial[Publication Type]) OR (controlled clinical trial[Publication Type])) OR (randomized[Title/Abstract])) OR (placebo[Title/Abstract])) OR ("Clinical Trials as Topic"[Mesh:NoExp])) OR (randomly[Title/Abstract])) OR (trial[Title/Abstract])) NOT (("Animals"[Mesh]) NOT ("Humans"[Mesh]))	1,660,993
#6	(#1 OR #2) AND #3 AND #4 AND #5	209

**Embase (September 16, 2023)**		
**Number**	**Searches**	**Results**
#1	'alteplase'/exp OR 'alteplase':ab,kw,ti OR 'actilyse':ab,kw,ti OR 'recombinant tissue plasminogen activator':ab,kw,ti OR 'rt pa'	26,211
#2	'tenecteplase'/exp OR 'tnk-tpa':ab,kw,ti OR 'tenecteplase':ab,kw,ti OR 'metalyse':ab,kw,ti OR 'tnkase':ab,kw,ti	3,482
#3	'ischemic stroke'/exp OR 'ischemic strokes':ab,kw,ti OR 'ischemic stroke':ab,kw,ti OR 'stroke, ischemic':ab,kw,ti OR 'ischaemic stroke':ab,kw,ti OR 'ischaemic strokes':ab,kw,ti OR 'stroke, ischaemic':ab,kw,ti OR 'cryptogenic ischemic stroke':ab,kw,ti OR 'cryptogenic ischemic strokes':ab,kw,ti OR 'ischemic stroke,	137,470

	cryptogenic':ab,kw,ti OR 'stroke, cryptogenic ischemic':ab,kw,ti OR 'cryptogenic stroke':ab,kw,ti OR 'cryptogenic strokes':ab,kw,ti OR 'stroke, cryptogenic':ab,kw,ti OR 'cryptogenic embolism stroke':ab,kw,ti OR 'cryptogenic embolism strokes':ab,kw,ti OR 'embolism stroke, cryptogenic':ab,kw,ti OR 'stroke, cryptogenic embolism':ab,kw,ti OR 'wake-up stroke':ab,kw,ti OR 'stroke, wake-up':ab,kw,ti OR 'wake up stroke':ab,kw,ti OR 'wake-up strokes':ab,kw,ti OR 'acute ischemic stroke':ab,kw,ti OR 'acute ischemic strokes':ab,kw,ti OR 'ischemic stroke, acute':ab,kw,ti OR 'stroke, acute ischemic':ab,kw,ti OR 'cerebral arterial thrombosis':ab,kw,ti OR 'ischemic cerebral apoplexy':ab,kw,ti	
#4	'thrombectomy'/exp OR 'embolectomy'/exp OR 'Thrombectomy':ab,kw,ti OR 'Embolectomy':ab,kw,ti OR 'Thrombectomy':ab,kw,ti OR 'Thrombectomies':ab,kw,ti OR 'Percutaneous Aspiration Thrombectomy':ab,kw,ti OR 'Aspiration Thrombectomies, Percutaneous':ab,kw,ti OR 'endovascular thrombectomy':ab,kw,ti OR 'mechanical thrombectomy':ab,kw,ti OR 'Embolectomies':ab,kw,ti	50,163
#5	'randomized controlled trial'/exp OR 'controlled clinical trial'/exp OR 'randomization'/exp OR 'placebo'/exp OR 'randomized controlled trial':ti,ab,kw OR 'rct':ti,ab,kw OR 'controlled clinical trial':ti,ab,kw OR 'random allocation':ti,ab,kw OR 'placebos':ti,ab,kw OR 'random':ti,ab,kw OR 'randomization':ti,ab,kw OR 'trial':ti,ab,kw NOT ('animals':ti,ab,kw NOT 'humans':ti,ab,kw)	2,310,385
#6	(#1 OR #2) AND #3 AND #4 AND #5	529

**Web of Science (September 16, 2023)**		
**Number**	**Searches**	**Results**
#1	((TS=(recombinant tissue plasminogen activator)) OR TS=(alteplase)) OR TS=(rt-PA)	19,613

#2	(((TS=(tenecteplase)) OR TS=(TNK-tPA)) OR TS=(Metalyse)) OR TS=(TNKase)	1,599
#3	((((((((TS=(Ischemic Stroke*)) OR TS=(Ischaemic Stroke)) OR TS=(Ischaemic Strokes)) OR TS=(Cryptogenic Ischemic Stroke*)) OR TS=(Cryptogenic Stroke*)) OR TS=(Cryptogenic Embolism Stroke*)) OR TS=(Wake-up Stroke*)) OR TS=(Wake up Stroke)) OR TS=(cerebral arterial thrombosis)) OR TS=(ischemic cerebral apoplexy)	216,696
#4	((((((((TS=(Thrombectomy)) OR TS=(Embolectomy)) OR TS=(Thrombectomies)) OR TS=(Percutaneous Aspiration Thrombectomy)) OR TS=(Aspiration Thrombectomies, Percutaneous)) OR TS=(endovascular thrombectomy)) OR TS=(mechanical thrombectomy)) OR TS=(Embolectomies))	35,399
#5	((((((TS=(randomized controlled trial)) OR TS=(controlled clinical trial)) OR TS=(randomized)) OR TS=(placebo)) OR TS=(randomly)) OR TS=(trial)) NOT (TS=(Animals)) NOT TS=(Humans)	3,726,009
#6	(#1 OR #2) AND #3 AND #4 AND #5	1,028

**Cochrane Library (September 16, 2023)**		
**Number**	**Searches**	**Results**
#1	MeSH descriptor: [Tissue Plasminogen Activator] explode all trees OR (Tissue Plasminogen Activator):ti,ab,kw OR ("recombinant tissue plasminogen activator" OR "alteplase" OR "rt-PA"):ti,ab,kw	5,245
#2	MeSH descriptor: [Tenecteplase] explode all trees OR ("tenecteplase" OR "TNK-tPA" OR "Metalyse" OR "TNKase"):ti,ab,kw	511
#3	MeSH descriptor: [Ischemic Stroke] explode all trees OR ("Ischaemic Stroke" OR " Stroke, Ischaemic" OR " Ischaemic Strokes" OR " Stroke, Ischemic" OR " Ischemic Strokes" OR " Wake up Stroke" OR " Wake-up Strokes" OR " Wake-up Stroke" OR " Stroke, Wake-up" OR " Cryptogenic Strokes" OR " Cryptogenic Embolism Stroke" OR " Stroke,	13,583

	Cryptogenic Embolism" OR " Stroke, Cryptogenic" OR " Cryptogenic Embolism Strokes" OR " Ischemic Stroke, Cryptogenic" OR " Cryptogenic Ischemic Strokes" OR " Cryptogenic Stroke" OR " Stroke, Cryptogenic Ischemic" OR " Embolism Stroke, Cryptogenic" OR " Cryptogenic Ischemic Stroke" OR " Acute Ischemic Stroke" OR " Stroke, Acute Ischemic" OR " Acute Ischemic Strokes" OR " Ischemic Stroke, Acute" OR " cerebral arterial thrombosis " OR " ischemic cerebral apoplexy " OR "Ischemic Stroke"):ti,ab,kw	
#4	MeSH descriptor: [Thrombectomy] explode all trees OR MeSH descriptor: [Embolectomy] explode all trees OR ("Thrombectomy" OR "Embolectomy" OR "Thrombectomy" OR "Thrombectomies" OR "Percutaneous Aspiration Thrombectomy" OR "Aspiration Thrombectomies, Percutaneous" OR "endovascular thrombectomy" OR "mechanical thrombectomy" OR "Embolectomies"):ab,kw,ti	2,305
#5	MeSH descriptor: [Randomized Controlled Trial] explode all trees OR MeSH descriptor: [Controlled Clinical Trial] explode all trees OR MeSH descriptor: [Random Allocation] explode all trees OR MeSH descriptor: [Placebos] explode all trees OR ("Randomized Controlled Trial " OR "RCT" OR " Controlled Clinical Trial " OR " Random Allocation " OR " Placebos " OR " Random " OR " Randomization " OR " Trial"):ti,ab,kw NOT ("Animals" NOT "Humans"):ti,ab,kw	1,111,328
#6	(#1 OR #2) AND #3 AND #4 AND #5	313

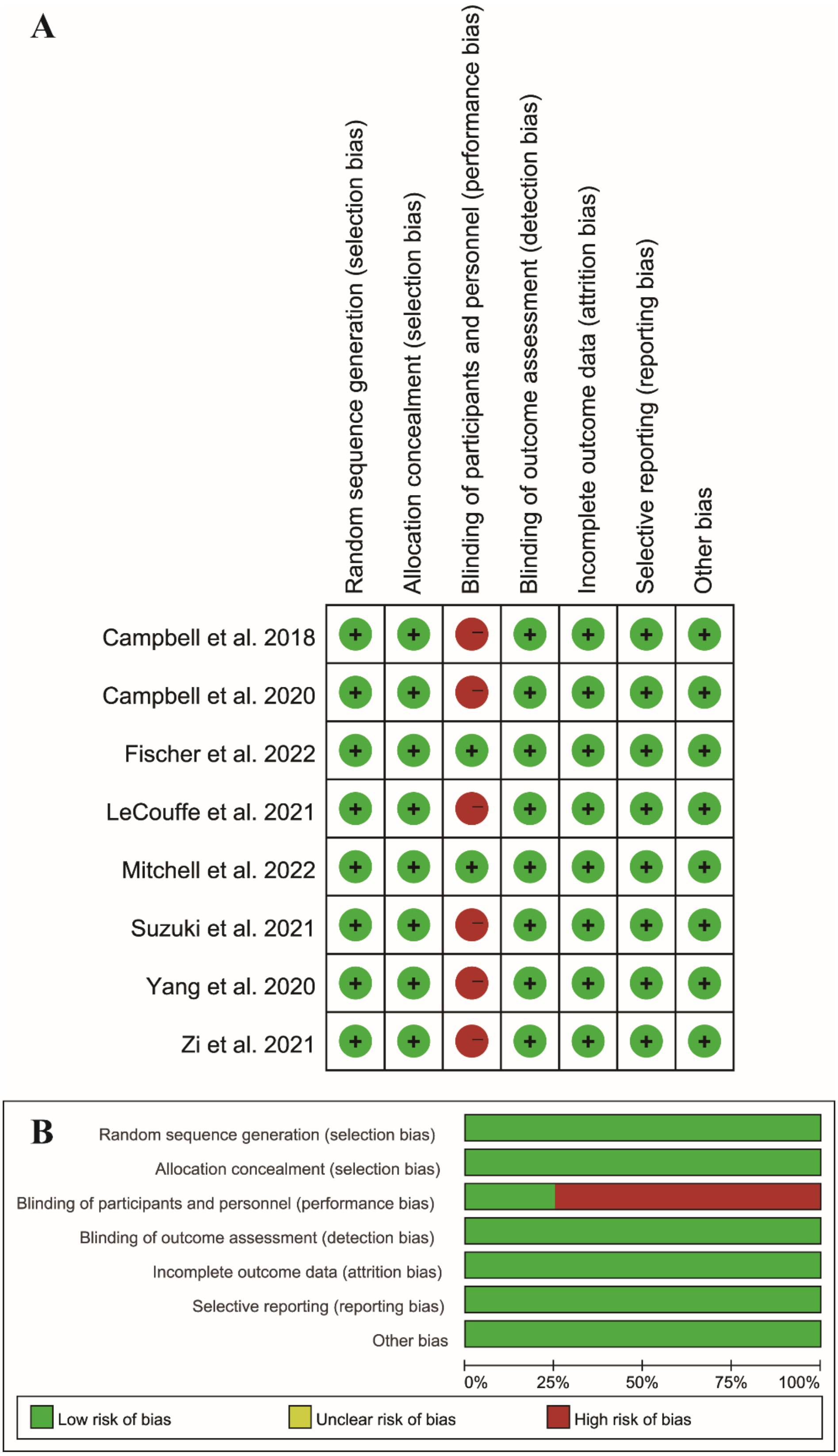

Fig. S1. Risk of bias of included studies. (A) Risk of bias summary: judgments about each bias item for each study; (B) Risk of bias summary graph.

A

Study or Subgroup	IVT+EVT Events	IVT+EVT Total	EVT alone Events	EVT alone Total	Weight	Risk Ratio M-H, Fixed, 95% CI
Fischer et al. 2022	135	207	114	201	20.2%	1.15 [0.98, 1.34]
LeCouffe et al. 2021	136	266	134	273	23.1%	1.04 [0.88, 1.23]
Mitchell et al. 2022	89	147	80	146	14.0%	1.10 [0.91, 1.35]
Suzuki et al. 2021	59	103	60	101	10.6%	0.96 [0.76, 1.22]
Yang et al. 2020	121	329	119	327	20.9%	1.01 [0.83, 1.24]
Zi et al. 2021	55	118	63	116	11.1%	0.86 [0.66, 1.11]
**Total (95% CI)**		**1170**		**1164**	**100.0%**	**1.04 [0.96, 1.12]**
Total events	595		570			

Heterogeneity: Chi² = 4.63, df = 5 (P = 0.46); I² = 0%

Test for overall effect: Z = 0.90 (P = 0.37)

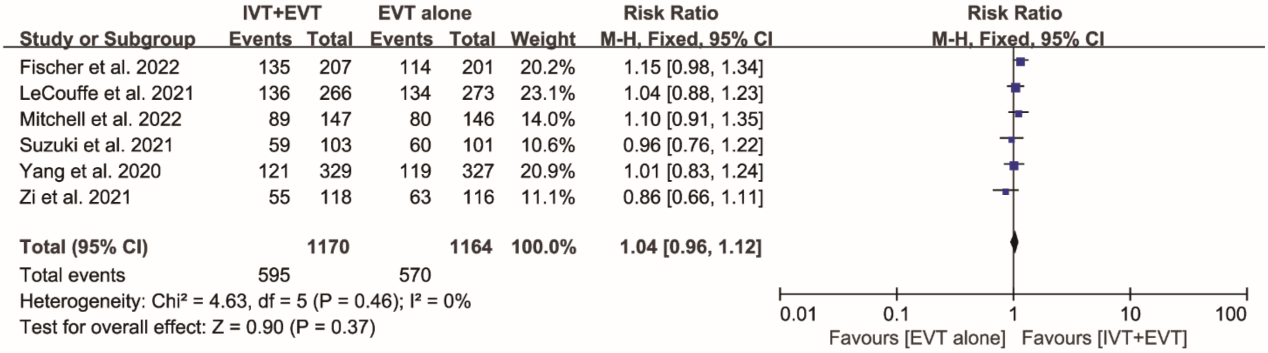

B

Study or Subgroup	IVT+EVT Events	IVT+EVT Total	EVT alone Events	EVT alone Total	Weight	Risk Ratio M-H, Fixed, 95% CI
LeCouffe et al. 2021	41	266	44	273	16.0%	0.96 [0.65, 1.41]
Mitchell et al. 2022	71	147	62	146	22.9%	1.14 [0.88, 1.46]
Suzuki et al. 2021	46	103	41	101	15.2%	1.10 [0.80, 1.51]
Yang et al. 2020	74	329	80	327	29.5%	0.92 [0.70, 1.21]
Zi et al. 2021	37	118	44	116	16.3%	0.83 [0.58, 1.18]
**Total (95% CI)**		**963**		**963**	**100.0%**	**0.99 [0.86, 1.13]**
Total events	269		271			

Heterogeneity: Chi² = 2.90, df = 4 (P = 0.58); I² = 0%

Test for overall effect: Z = 0.18 (P = 0.86)

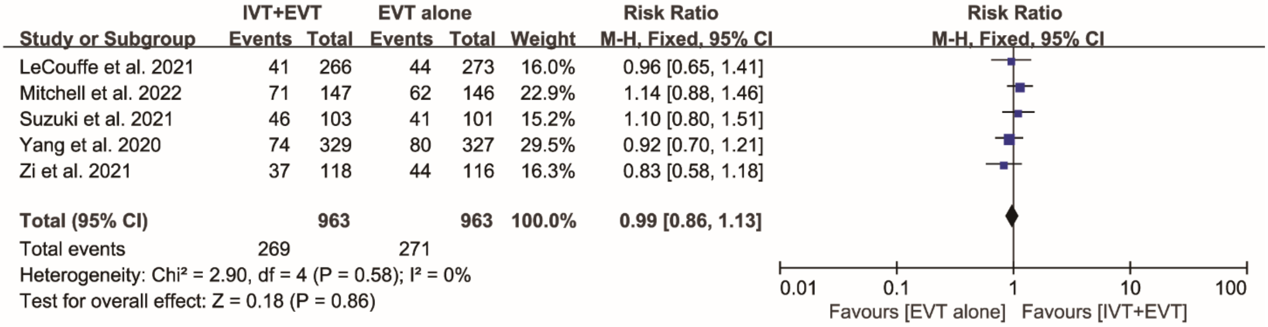

C

Study or Subgroup	IVT+EVT Events	IVT+EVT Total	EVT alone Events	EVT alone Total	Weight	Risk Ratio M-H, Fixed, 95% CI
Fischer et al. 2022	17	207	22	201	12.0%	0.75 [0.41, 1.37]
LeCouffe et al. 2021	42	266	56	273	29.7%	0.77 [0.54, 1.11]
Mitchell et al. 2022	24	147	22	146	11.9%	1.08 [0.64, 1.84]
Suzuki et al. 2021	9	103	8	101	4.3%	1.10 [0.44, 2.75]
Yang et al. 2020	62	329	58	327	31.3%	1.06 [0.77, 1.47]
Zi et al. 2021	21	118	20	116	10.8%	1.03 [0.59, 1.80]
**Total (95% CI)**		**1170**		**1164**	**100.0%**	**0.94 [0.78, 1.13]**
Total events	175		186			

Heterogeneity: Chi² = 2.76, df = 5 (P = 0.74); I² = 0%

Test for overall effect: Z = 0.65 (P = 0.51)

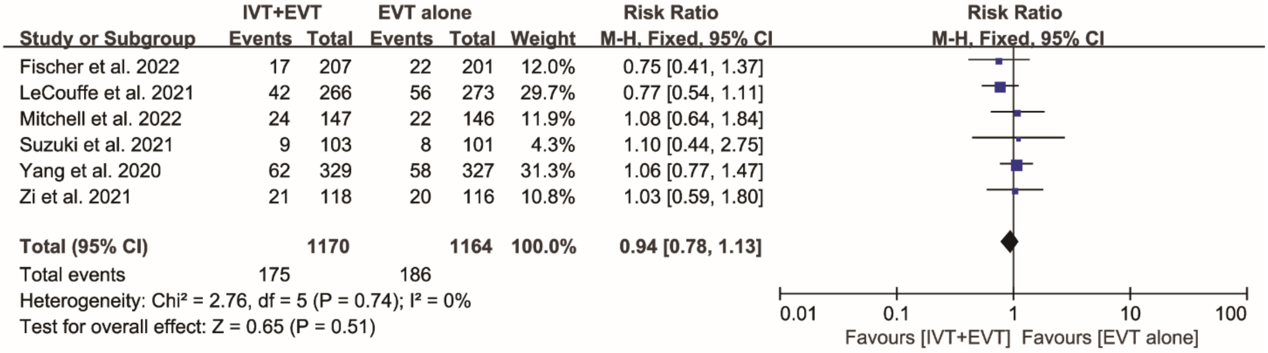

D

Study or Subgroup	IVT+EVT Events	IVT+EVT Total	EVT alone Events	EVT alone Total	Weight	Risk Ratio M-H, Fixed, 95% CI
Fischer et al. 2022	199	207	182	201	19.6%	1.06 [1.01, 1.12]
LeCouffe et al. 2021	196	236	192	244	20.0%	1.06 [0.97, 1.15]
Mitchell et al. 2022	130	146	127	143	13.6%	1.00 [0.92, 1.09]
Suzuki et al. 2021	96	103	91	101	9.8%	1.03 [0.95, 1.12]
Yang et al. 2020	267	316	243	306	26.2%	1.06 [0.99, 1.15]
Zi et al. 2021	102	117	100	113	10.8%	0.99 [0.89, 1.08]
**Total (95% CI)**		**1125**		**1108**	**100.0%**	**1.04 [1.01, 1.08]**
Total events	990		935			

Heterogeneity: Chi² = 3.08, df = 5 (P = 0.69); I² = 0%
Test for overall effect: Z = 2.45 (P = 0.01)

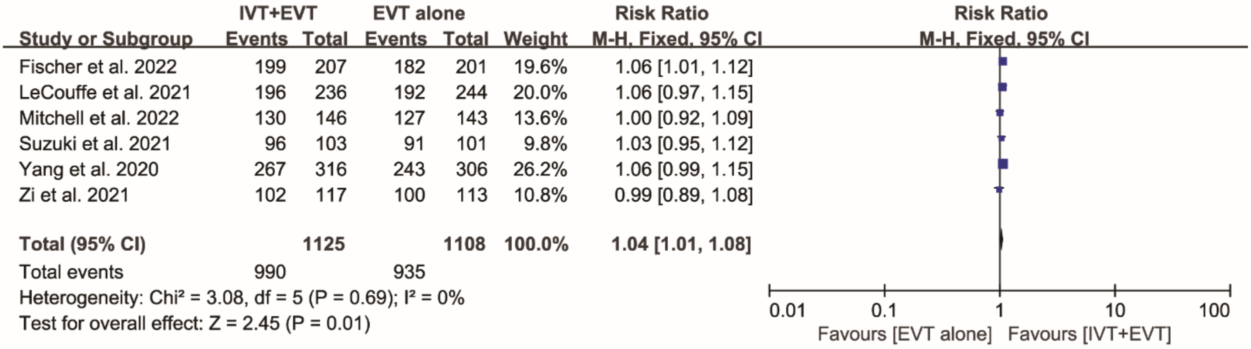

E

Study or Subgroup	IVT+EVT Events	IVT+EVT Total	EVT alone Events	EVT alone Total	Weight	Risk Ratio M-H, Fixed, 95% CI
Fischer et al. 2022	7	207	5	201	10.8%	1.36 [0.44, 4.21]
LeCouffe et al. 2021	14	266	16	273	33.6%	0.90 [0.45, 1.80]
Mitchell et al. 2022	1	147	2	146	4.3%	0.50 [0.05, 5.42]
Suzuki et al. 2021	8	103	6	101	12.9%	1.31 [0.47, 3.63]
Yang et al. 2020	20	329	14	327	29.9%	1.42 [0.73, 2.76]
Zi et al. 2021	5	117	4	115	8.6%	1.23 [0.34, 4.46]
**Total (95% CI)**		**1169**		**1163**	**100.0%**	**1.17 [0.80, 1.71]**
Total events	55		47			

Heterogeneity: Chi² = 1.49, df = 5 (P = 0.91); I² = 0%
Test for overall effect: Z = 0.80 (P = 0.42)

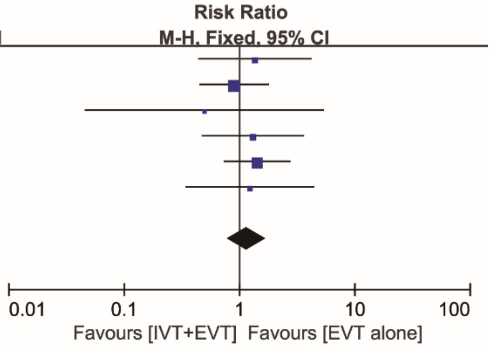

F

Study or Subgroup	IVT+EVT Events	IVT+EVT Total	EVT alone Events	EVT alone Total	Weight	Risk Ratio M-H, Fixed, 95% CI
Fischer et al. 2022	69	207	59	201	16.6%	1.14 [0.85, 1.52]
LeCouffe et al. 2021	85	239	89	248	24.2%	0.99 [0.78, 1.26]
Mitchell et al. 2022	32	147	31	146	8.6%	1.03 [0.66, 1.59]
Suzuki et al. 2021	52	103	34	101	9.5%	1.50 [1.07, 2.09]
Yang et al. 2020	139	329	123	327	34.2%	1.12 [0.93, 1.36]
Zi et al. 2021	38	117	25	115	7.0%	1.49 [0.97, 2.31]
**Total (95% CI)**		**1142**		**1138**	**100.0%**	**1.15 [1.02, 1.28]**
Total events	415		361			

Heterogeneity: Chi² = 5.65, df = 5 (P = 0.34); I² = 12%
Test for overall effect: Z = 2.35 (P = 0.02)

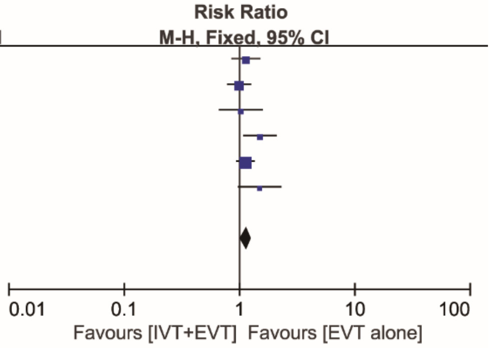

Fig. S2. Forest plot of subgroups analysis of outcomes. (A) functional independence (mRS 0–2) at 90 days; (B) excellent outcome (mRS 0–1) at 90 days; (C) all-cause mortality at follow-up; (D) successful reperfusion (TICI 2b–3) after the end of EVT; (E) symptomatic intracranial hemorrhage; (F) any intracranial hemorrhage.

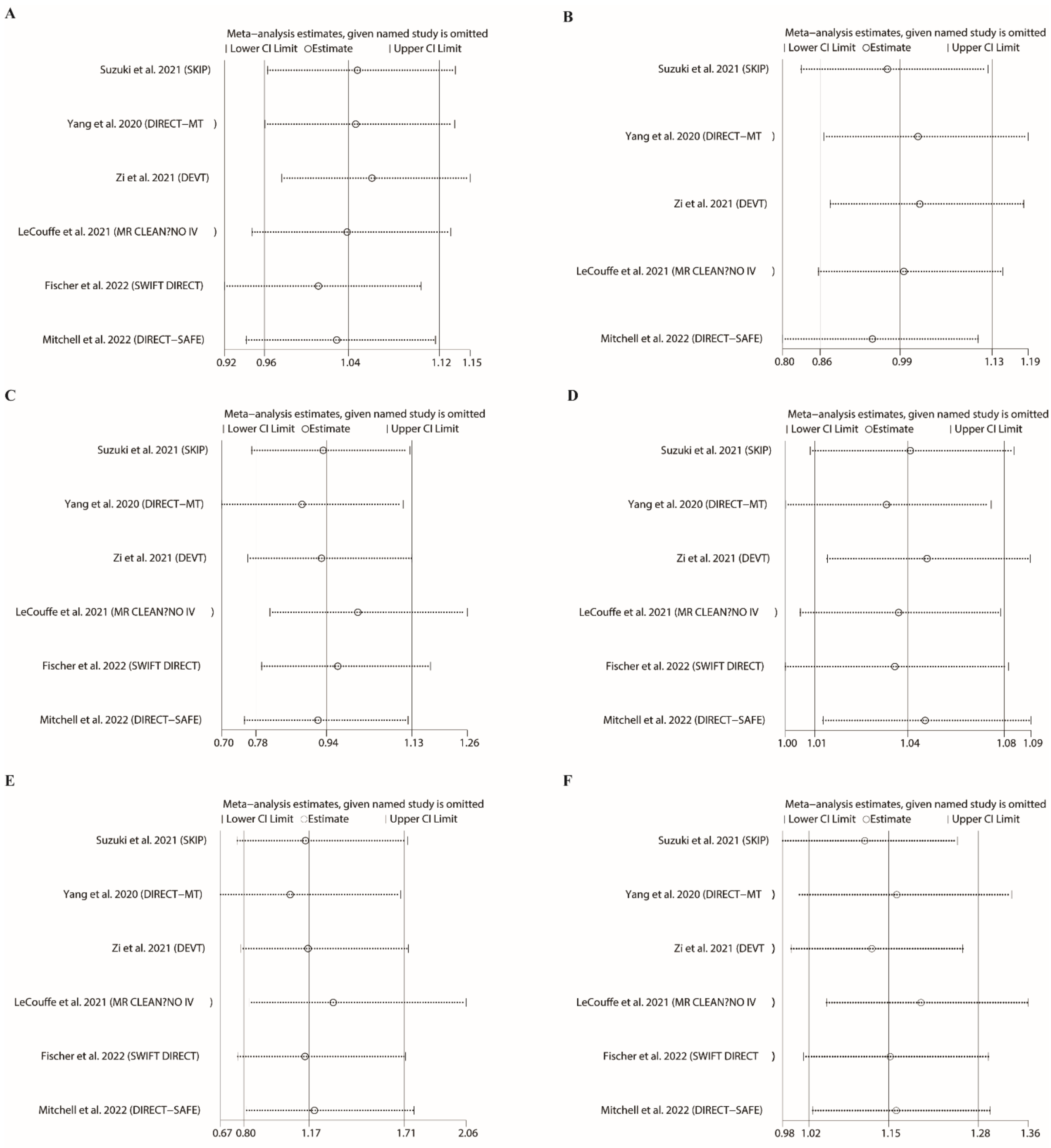

Fig. S3. Sensitivity analysis with leave-one-out method for outcomes. (A) functional independence (mRS 0–2) at 90 days; (B) excellent outcome (mRS 0–1) at 90 days; (C) all-cause mortality at follow-up; (D) successful reperfusion (TICI 2b–3) after the end of EVT; (E) symptomatic intracranial hemorrhage; (F) any intracranial hemorrhage.

Supplementary Table 3. Meta-regression analysis of outcomes.

Variables	mRS(0-2)	mRS(0-1)	All-cause mortality	TICI	sICH	aICH
	P value	*P* value	*P* value	*P* value	*P* value	*P* value
Age	0.864	0.606	0.516	0.724	0.945	0.266
Male(%)	0.442	0.438	0.352	0.692	0.438	0.544
NIHSS score	0.834	0.779	0.848	0.376	0.402	0.24
mRS 0	0.186	0.274	0.42	0.47	0.812	0.768
mRS 1	0.358	0.981	0.413	0.408	0.598	0.149
mRS 2	/	/	/	/	/	/
ASPECTS	0.607	0.575	0.772	0.71	0.613	0.14
Cardioembolic occlusion(%)	0.46	0.713	0.349	0.564	0.557	0.157
Large-artery occlusion(%)	0.42	0.871	0.913	0.345	0.778	0.374
Undetermined or other	0.409	0.861	0.368	0.48	0.565	0.153
Stroke onset to hospital arrival	/	/	/	/	/	/
Stroke onset to randomization	0.48	0.529	0.229	0.609	0.439	0.461
Stroke onset to initiation of intravenous thrombolysis	0.92	0.422	0.934	0.583	0.542	0.696
Randomization–to–rt-PA time		0.714	0.95	0.901	0.956	0.369
From hospital admission to rt-PA	0.364	0.791	0.831	0.616	0.556	0.556
Initiation of intravenous thrombolysis to arterial puncture	0.891	0.39	0.416	0.232	0.869	0.663
Mean time from symptom onset to MT	0.595	0.8	0.406	0.808	0.526	0.533
Randomization-to-puncture time	0.59	0.891	0.968	0.926	0.967	0.353
From hospital admission to groin puncture	0.492	0.973	0.275	0.258	0.602	0.283
Procedure length						
Internal carotid artery(%)	0.553	0.661	0.39	0.661	0.372	0.246
Basilar artery(%)						
M1(%)	0.621	0.347	0.672	0.6	0.809	0.783
M2(%)	0.878	0.267	0.833	0.958	0.583	0.438
Middle cerebral artery(%)	0.645	0.914	0.297	/	0.452	0.358
Tandem lesion(%)	0.424	0.652	0.563	0.84	0.785	0.973
AF(%)	0.249	0.469	0.431	0.808	0.445	0.114
Diabetes mellitus(%)	0.411	0.393	0.482	0.469	0.687	0.636

Hypertension(%)	0.393	0.562	0.304	0.338	0.512	0.169
Dyslipidemia(%)	0.567	0.555	0.31	0.983	0.629	0.78
Past stroke(%)	0.291	0.567	0.847	0.366	0.472	0.643
Past cardiovascular disease(%)	/	/	/	/	0.873	/
Anticoagulant agent(%)	0.691	0.604	0.457	0.76	0.636	0.299
Antiplatelet agent(%)	0.743	0.914	0.406	0.957	0.513	0.521
Current smoking(%)	0.908	0.513	0.969	0.873	0.918	0.374
Serum glucose(%)	0.628	0.4	0.713	0.763	0.864	0.2
Systolic(%)	0.998	0.376	0.473	0.99	0.71	0.673
Diastolic(%)	/	/	/	/	/	/

Supplementary Table 4. The results of Begg and Egger.

	Begg	Egger
	P value	*P* value
mRS(0-2）	**0.024**	**0.023**
mRS(0-1）	0.462	0.354
All-cause mortality	0.707	0.879
TICI	0.133	0.137
sICH	0.452	0.555
aICH	0.707	0.352

Note: aICH, any intracranial hemorrhage; mRS, modified Rankin Scale; sICH, symptomatic intracranial hemorrhage; TICI, Thrombolysis in Cerebral Ischemia.

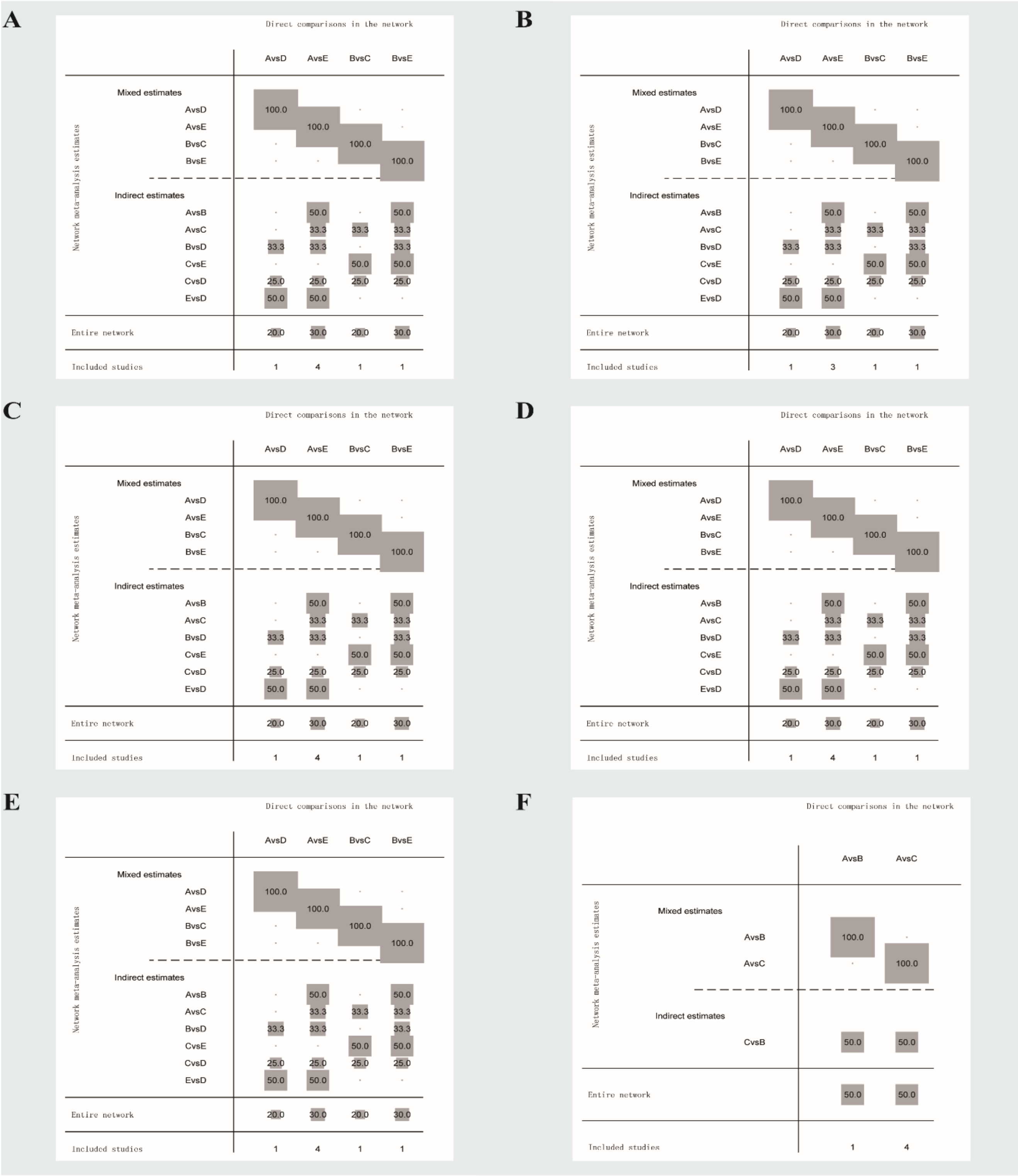

Fig. S4. Contribution plot of outcomes. (A) functional independence (mRS 0–2) at 90 days; (B) excellent outcome (mRS 0–1) at 90 days; (C) all-cause mortality at follow-up; (D) successful reperfusion (TICI 2b–3) after the end of EVT; (E) symptomatic intracranial hemorrhage; (F) any intracranial hemorrhage.
